# A Data Analytics/Big Data Framework for Advanced Metering Infrastructure Data

**DOI:** 10.3390/s21165650

**Published:** 2021-08-22

**Authors:** Jenniffer S. Guerrero-Prado, Wilfredo Alfonso-Morales, Eduardo F. Caicedo-Bravo

**Affiliations:** School of Electrical and Electronics Engineering, Faculty of Engineering, Universidad del Valle, Calle 13 #100-00, Cali P.O. Box 25360, Colombia; wilfredo.alfonso@correounivalle.edu.co (W.A.-M.); eduardo.caicedo@correounivalle.edu.co (E.F.C.-B.)

**Keywords:** advanced metering infrastructure, AMI data, big data, data analytics, SGAM, smart cities, smart grids, smart meter

## Abstract

The Advanced Metering Infrastructure (AMI) data represent a source of information in real time not only about electricity consumption but also as an indicator of other social, demographic, and economic dynamics within a city. This paper presents a Data Analytics/Big Data framework applied to AMI data as a tool to leverage the potential of this data within the applications in a Smart City. The framework includes three fundamental aspects. First, the architectural view places AMI within the Smart Grids Architecture Model-SGAM. Second, the methodological view describes the transformation of raw data into knowledge represented by the DIKW hierarchy and the NIST Big Data interoperability model. Finally, a binding element between the two views is represented by human expertise and skills to obtain a deeper understanding of the results and transform knowledge into wisdom. Our new view faces the challenges arriving in energy markets by adding a binding element that gives support for optimal and efficient decision-making. To show how our framework works, we developed a case study. The case implements each component of the framework for a load forecasting application in a Colombian Retail Electricity Provider (REP). The MAPE for some of the REP’s markets was less than 5%. In addition, the case shows the effect of the binding element as it raises new development alternatives and becomes a feedback mechanism for more assertive decision making.

## 1. Introduction

One of the pillars of Smart Cities is the intensive use of information-based technologies. Thus, big data and data analytics have become robust tools that support the development of applications for actors involved in them. One of the most important actors is Smart Grids, which enable data harvesting to implement an evolved and more efficient electrical network. Adopting Advanced Metering Infrastructure (AMI) is geared toward promoting tools available to quantify and measure the energy flow throughout the grid.

This infrastructure not only acts to provide information to the utility but it also enables the customer as a stakeholder in the energy value chain. AMI data represent a source of information in real time not only on electricity consumption but also potentially on population behaviors, such as concentrations of people, population migration, demographic trends, and economic changes in various sectors of the population, among others [1].

The latest study published by Berg Insight on smart meter markets stated that the three markets that lead the way in smart energy meter installation are Asia–Pacific, Europe, and the US. Studies expect that, in 2024, the Asia–Pacific market (i.e., China, Japan, South Korea, India, Australia, and New Zealand) will reach 975 million smart meters, USD 142.8 million [2], and Europe around 223 million [3]. Considering that a smart meter can record information at minute intervals, the amount of information available is massive. Precisely this imminent arrival of large volumes of information means that the areas of Big Data and Data Analytics are required tools to explore and analyze such data.

In this regard, several authors have worked on different applications that seek to extract value from these raw data available from smart meters or AMI assets. Among the most common topics that involve advanced analytic techniques and big data in AMI data, we can highlight:

- AMI data processing tools and their integration with emerging technologies. This group includes work related to platforms for storing and running analytic using different applications, including Hadoop, MATLAB, MADlib, System C, Hive, and Spark Streaming [4,5,6]. Some authors have also explored performance evaluation platforms [7] as well as some emerging technologies, such as cloud computing, real-time data processing [8], and fog computing [9]. In addition, we can also include authors dedicated to developing methodological approaches to process AMI data [10,11].
- Load Profile Identification. This is one of the most common applications developed with AMI data. It implements algorithms to identify customer’s consumption profiles [12]. Some tools used for this type of applications are clustering algorithms [13], artificial neural networks [14], self-organizing maps (SOM) [15], or support vector machines (SVM) [16]. Some authors have even implemented applications that reach the level of load disaggregation to evaluate consumption patterns [17,18,19]. In this sense, there are two major approaches, namely Intrusive Load Monitoring (ILM) and Non-Intrusive Load Monitoring (NILM). ILM requires the installation of additional measurement equipment, increasing implementation costs, while NILM is presented as an approach that requires more intensive analytical methods, with a lower hardware investment [20,21,22].
- Load Forecasting. This application seeks to predict the energy demand of customers. It has become one of the most studied since it improves the planning processes into the utilities’ operation. Some authors have used approaches, such as knowledge discovery from database methodologies (KDD) [23], machine learning [24,25], evolutionary algorithms [26,27], clustering [28], and deep residual networks [29].
- Demand Response Programs. This is one of the applications with the highest potential in Smart Grids because it enables customer participation in the value chain. These programs intentionally seek to modify a consumer’s consumption pattern to reduce consumption peaks, as manifested in the utility demand curves. Some authors have implemented advanced analytical techniques focused on this type of application [30,31] and evaluated their effectiveness [32].
- Loss detection. This generally concentrates on the detection of non-technical losses and represents cases with direct monetization of the application. In this regard, several authors have proposed to use different data analysis techniques, including Extreme Learning Machines, Genetic Support Vector Machines, Boolean Rules, Fuzzy Logic, SVM [33], or spectral analysis of periodic patterns [34].

There are currently several studies on Big Data and Data Analytics applications for AMI data. However, it is evident that fractional developments do not incorporate a global integration of architectural components of the data life cycle or consider a complete methodology. On the one hand, some Big Data and Data Analytic methodologies have already been defined [35]. On the other hand, there are architectures to develop Smart Grids in which the AMI systems are framed [36].

For instance, the work developed in [37] combined both areas in a framework, but only as an architectural view (Where?, Using what?) and not as a methodological one (How?). Most of the developments found in the literature are focused on the application of methods but leave aside some cross-cutting architecture components, such as information security and privacy, data governance, information integration and sharing, platform scalability, the variability of the requirements, and the data sources over time. Based on the above, two main focuses are evident: AMI as the one for Smart Grids and Data Analytics/Big Data as tools to give value to the data generated from the AMI deployment.

We have purposely preferred to separate the terms Data Analytics and Big Data. The first one is about the raw data transformation into usable knowledge through different algorithms and techniques [7]. The second term refers to the characteristic of the data itself to be processed (volume, velocity, and variety). Thus, depending on the data, a Data Analytics application may or may not be considered “Big.” The difference is that Big Data applications require much more complex processing platforms due to the nature of the source data. Even so, several authors prefer to use the term Big Data Analytics to work with one or the other approach together [38,39,40].

Based on these concepts, in this work, we aim to present a Data Analytics/Big Data framework for AMI data using human expertise and skills as a binding element. The human expertise incorporates architectures (Where?) and methods (How?) to transform and give value to the AMI data. Such transformation brings profits to the Smart Grid, the company, and its customers, in addition to the evidenced requirements, which are not only technological but also related to human training and skills.

The following section presents the proposed framework and describes its components. We validated this framework through two case studies. The first case about the analysis of electricity consumption data from smart meters in London from 2011 to 2014 was already reported in [41]. Section 3 presents the second one, which implements each component of the framework for a load forecasting application in a Colombian Retail Electricity Provider (REP). Finally, Section 4 presents the main conclusions, highlights, and future directions.

## 2. The Data Analytics/Big Data Framework for Ami Data

This section presents an implementation framework to join tools of both niches: AMI data and Data Analytics/Big Data, whether data is considered “Big” or not. The framework fits them crosswise by incorporating a binding element. This section describes these three major components. The first explicitly depicts AMI, its role, and deployment framed in the Smart Grids (SGAM) as an architectural view. The second major component involves the evolution process that AMI data has to undergo to generate value at the business level in SGAM as a methodological view. Finally, we present the binding element between the two previous components, i.e., human expertise and skills, which allows understanding the results previously obtained better.

### 2.1. Architecture View: Advanced Metering Infrastructure in the Smart Grid

**Figure 1:**
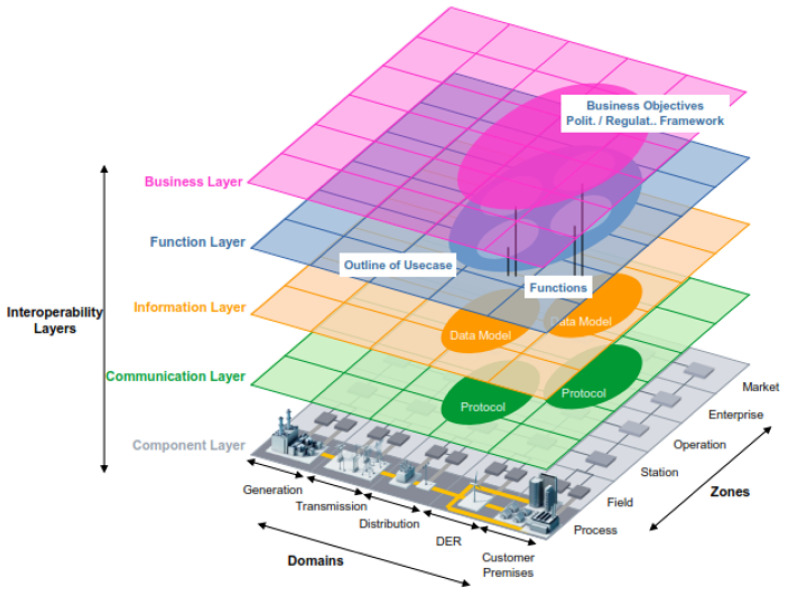
The Smart Grid Architecture Model (SGAM).

The first architectural element considered for the proposed framework is the same used for Smart Grids. In this regard, several authors propose some architectures to establish a common framework to develop Smart Grids applications [42,43,44]. However, the architecture presented by CENELEC has become a benchmark in the context of Smart Grids around the world [36]. Figure 1 depicts this Smart Grid Architecture Model, well known as SGAM.

SGAM took its inspiration from the model proposed by the GridWise Architecture Council. The basis of this model is the concept of interoperability, which is considered a key enabler of Smart Grids [45]. Interoperability suggests the ability of two systems, from the same or different manufacturer, to exchange information and use it correctly for the operation of a process [46]. Based on this model, CENELEC grouped the eight proposed categories by the GridWise Council into five interoperability layers, as shown in Figure 1. This grouping aims to introduce an architecture that facilitates the implementation of use cases applicable to the context of Smart Grid. Below we describe each interoperability layer briefly.

- Business Layer: refers to the global vision from a business view. This layer supports decision-making oriented to new business models, business cases, new market models, among others.
- Function layer: refers to functions and services, as well as the relationships between them. Since the business layer implies functions, they must be considered independently of the actors and components to achieve the proposed functionalities.
- Information Layer: refers to the information exchanged between devices, functions, and services. It also contemplates data models representing the semantics of the information that flows at each network stage.
- Communication Layer: refers to the protocols and mechanisms for the interoperable exchange of information between the Smart Grid components.
- Component Layer: refers to all the physical components present in the context of Smart Grids, e.g., actors, applications, power assets, protection devices, and network infrastructure.

In addition to the interoperability layers, the CENELEC architecture considers the Smart Grid Plane, located at the bottom of Figure 1. This plane represents the interaction between the processes (physical domains) of the electrical energy conversion chain and management viewpoints (hierarchical zones or levels).

- Bulk Generation: represents the processes of energy generation in large quantities, such as hydroelectric plants, nuclear plants, and solar and wind farms.
- Transmission: represents the infrastructure arranged to transport electricity over long distances.
- Distribution: refers to the infrastructure arranged to distribute energy to the end-user.
- Distributed Electrical Resources (DER): refer to small-scale power generation technologies connected directly to the distribution network.
- Customer Premises: refers to all the facilities of clients who may be electricity consumers or even producers. Premises include industrial, commercial, and home facilities.

In addition to the domains, the SGAM zones represent the different hierarchical levels of electrical system management. The main objective of the zones is to establish functional separation in the power system [47]. These zones are:

- Process: includes the physical, chemical, or spatial transformation of energy and all the equipment involved in these processes.
- Field: refers to equipment to protect, control, and monitor power system processes.
- Station: represents data concentration elements for an area just as supervision and automation systems for plants or substations.
- Operation: refers to power system control operations for each domain.
- Enterprise: includes the organizational and commercial processes of each utility, service provider, or energy trader. These processes include asset management, workforce management, logistics, and staff training, among others.
- Market: considers all the operations that can take place in the wholesale energy market, retail energy markets, or the spot energy market.

In summary, the architecture integrates three core viewpoints: layers, zones, and domains. These three components form the Smart Grid Architecture Model (SGAM). Since the focus of this paper is AMI data, this section maps the deployment of AMI components over SGAM. In this regard, the work from [43] describes the requirements for AMI using SGAM. This description pictures from the business layer to the component layer.

**Figure 2:**
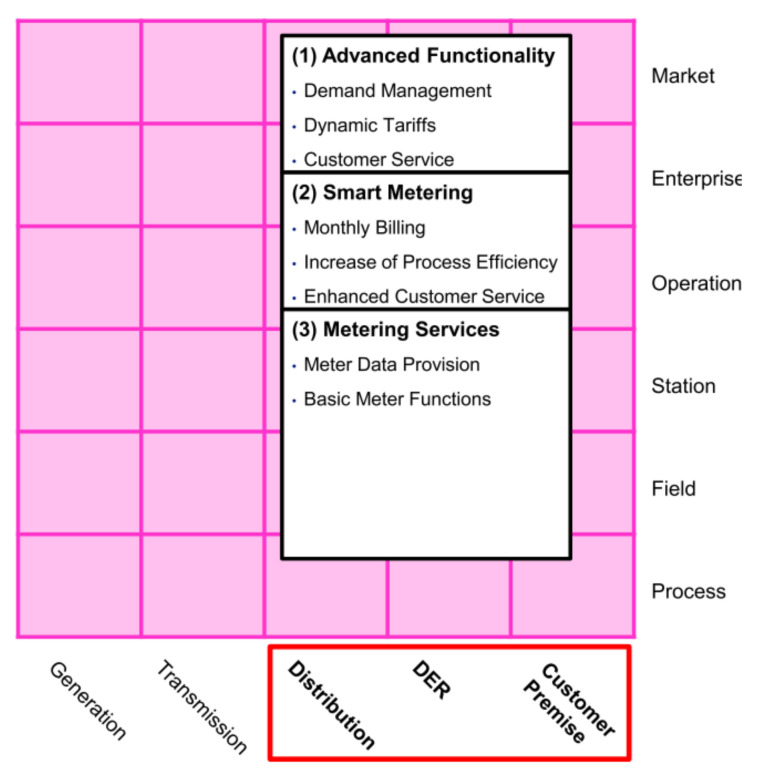
AMI business layer mapped over SGAM.

**Figure 3:**
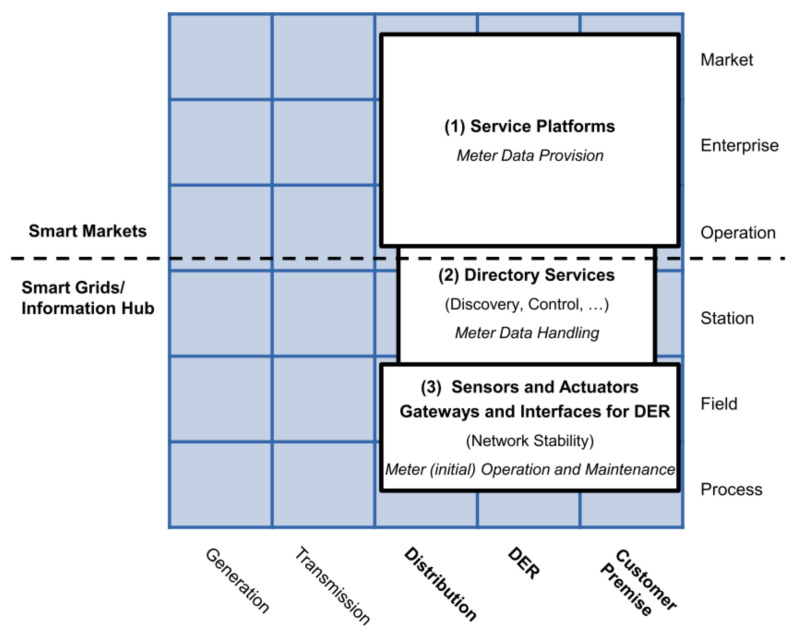
AMI function layer mapped over SGAM.

**Figure 4:**
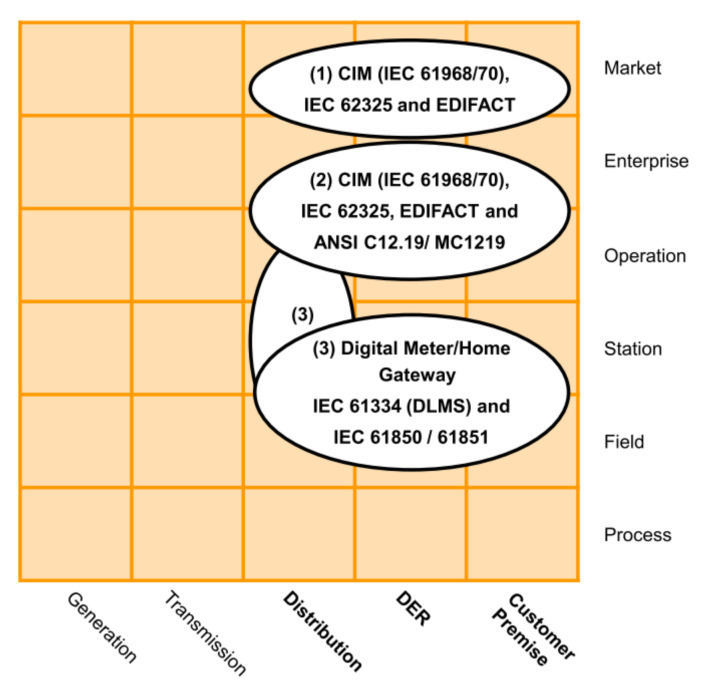
AMI information layer mapped over SGAM.

**Figure 5:**
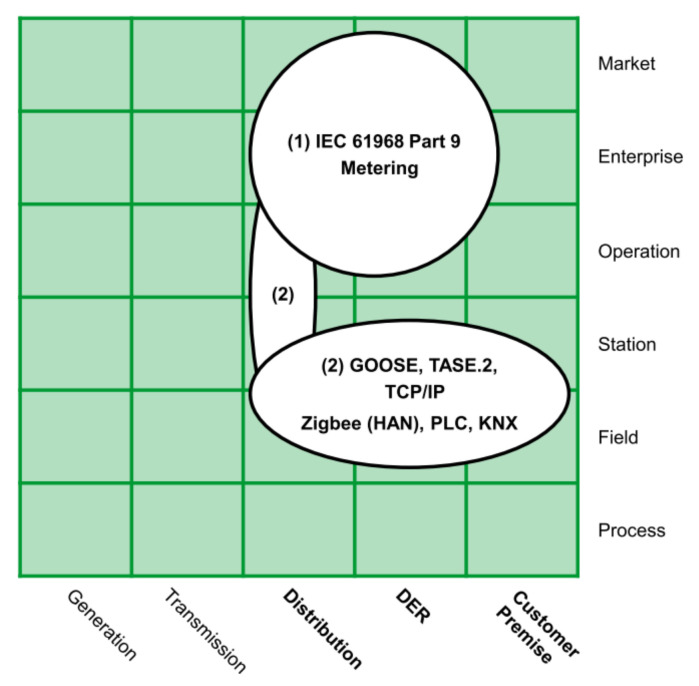
AMI communication layer mapped over SGAM.

**Figure 6:**
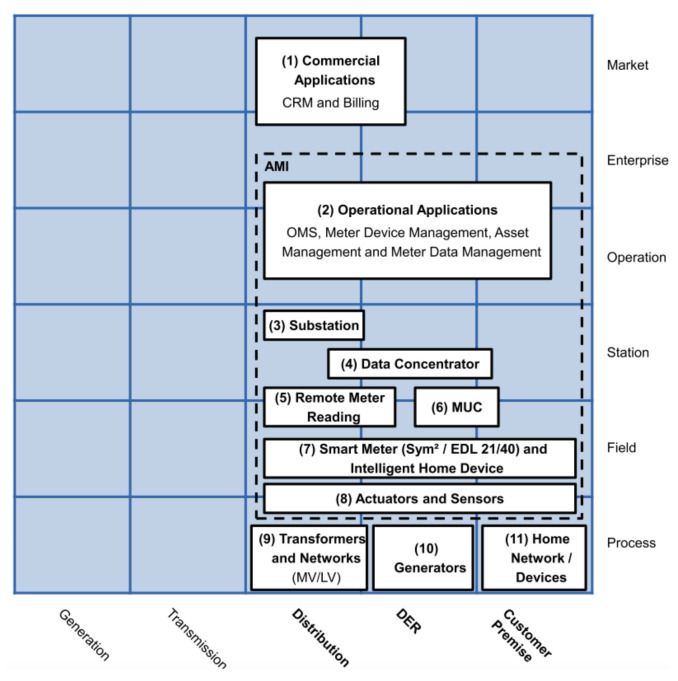
AMI component layer mapped over SGAM.

- **AMI business layer**
  This layer depicted in Figure 2 summarizes the AMI’s goals for a Smart Grid. These goals can involve three business functions that frame the general objectives of AMI: metering services, smart metering, and advanced functionality [43]: metering services refer to the primary measurement capabilities expected from AMI, i.e., at least minute interval energy measurements; smart metering refers to extended functions to gather data, e.g., billing and aggregated or detailed metering data; and finally advanced functionalities are related to extended goals, like dynamic tariffs and demand management.
- **AMI function layer**
  This layer depicted in Figure 3 defines the necessary functions; generally, Information Technology (IT) functions to achieve the goals established in the business layer. The layer presents three functional blocks: service platforms, directory services, and sensors or actuators. Service platforms are the metering data preparation for different roles, like the distribution network operator, DER operator, or customer. Directory service relates to the functions of controlling and accessing measuring devices. Sensors and actuator gateways refer to the interfaces for accessing and controlling such devices.
  This layer may be the most important for its role as a data provider for different applications. It involves the distribution, DER, and customer premises domains. It also covers the zones of operation, enterprise, and market.
  The German Federal Network Agency coined two terms in this context: Smart Grids and Smart Markets. Here, Smart grids refer to the operation of the network and its service provision infrastructure. Concerning IT functions defined in the function layer, these components act as an information hub. Smart Markets refer to instances outside the physical infrastructure of the network to trade services among market participants according to the available capacity of the network [48].
- **AMI information layer**
  The IEC Seamless Integration Architecture (SIA) was defined by the IEC Technical Committee 57 “Power Systems Management” (TC 57) [47]. Into SIA, the Common Information Model (CIM) serves as an information model for all entities participating in the market [49]. According to [43], the AMI information layer presented in Figure 4 contains three standard groups that define information models.
  The first group contains market-related standards and regulatory data formats. The second group includes relevant standards for the company and operation areas. The third group considers standards focused on the integration and control of measurement devices in the field zone.
- **AMI communication layer**
  This layer presented in Figure 5 includes the protocols for the transport of information between the different instruments of the measurement infrastructure.
  The authors in [43] identified two groups: the first one is related to protocols for data exchange in business and market zones, such as IEC 61968-9; and the second group involves protocols focused on the operation, station, and field zones, e.g., Zigbee or Goose.
- **AMI component Layer**
  This layer presented in Figure 6 is the lowest level of SGAM. This layer implements the requirements of the previous layers.
  This one presents two main groups. The first group represents the AMI core elements and includes the operating platforms and the technical equipment, such as information and communication technologies. The second group refers to secondary components related to AMI deployment, such as commercial applications or transformers and network equipment.

**Figure 7:**
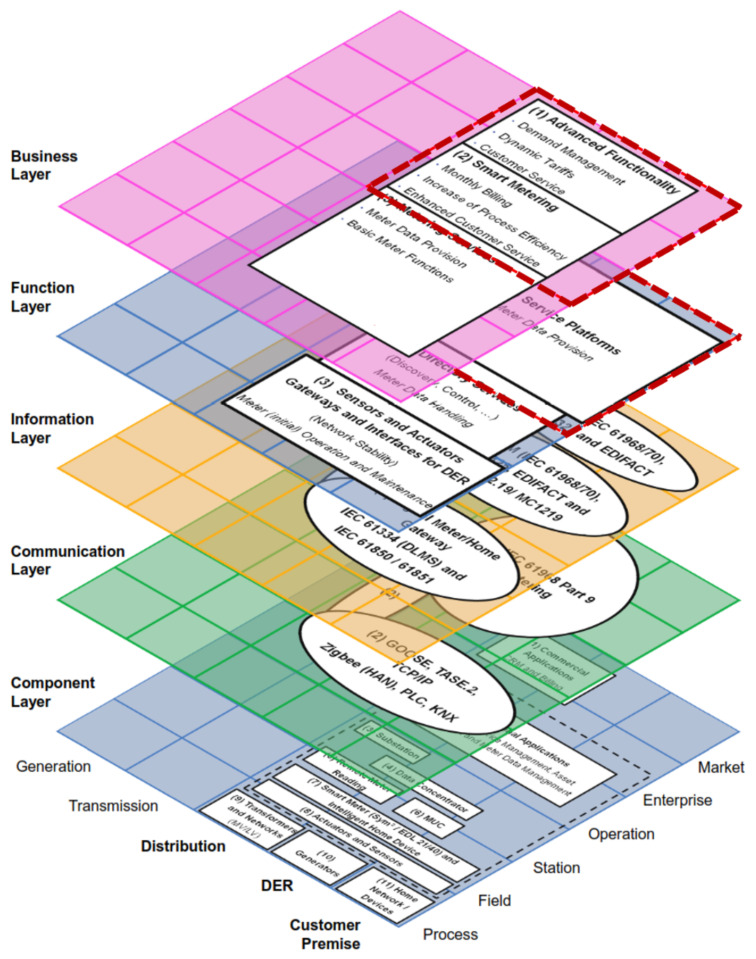
AMI components over SGAM.

Figure 7 groups together all the layers described above. This figure combines the layers from Figure 2, Figure 3, Figure 4, Figure 5 and Figure 6 in the standard SGAM representation [36], based on the work presented in [43]. The figure presents in red dotted line the SGAM layers, zones, and domains involved in the specification of our Data Analytics/Big Data framework for AMI data.

The SGAM business layer defines AMI’s goals for Smart Grids. These goals can include the basic functionalities of the smart metering system to the implementation of dynamic tariffs, billing, and demand management, among others. The function layer describes the platforms and services that fulfill the necessary functions to meet these goals. Thus, this layer offers metering data provision: customer databases, consumption information from each meter (power active or reactive energy), tariff schemes, and system events (interruptions, failures, connections, and disconnections), among others.

The analysis starts with the data coming from the service platforms, which are in charge of the metering data provision. In addition, the communication protocols, the data models, and the devices from which the information is coming from do not matter since the framework does not interfere with SGAM’s lower interoperability layers.

### 2.2. The Methodology View: Dikw Hierarchy and Nist Model

#### 2.2.1. The Dikw Hierarchy

In 1989 Russel Ackoff first proposed the wisdom concept as a hierarchical structure to define the evolution of an entity from its data character to a higher level [50]. In his work, the hierarchy included the following stages: data, information, knowledge, understanding, and wisdom. However, several authors in the literature prefer to place the understanding as part of the knowledge [51].

**Figure 8:**
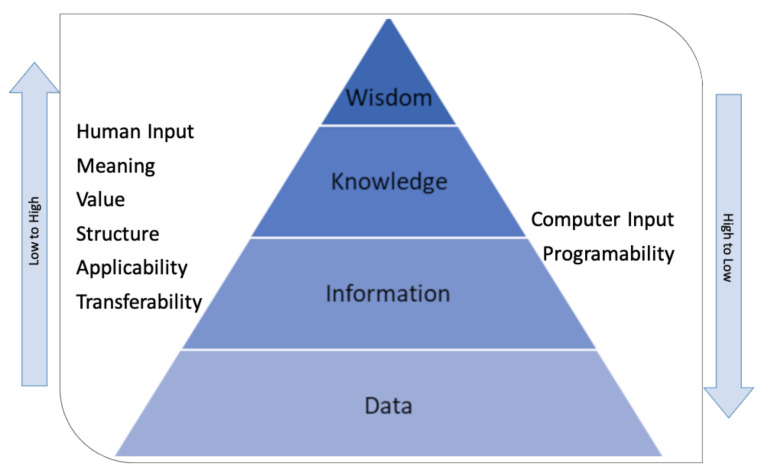
Data, Information, Knowledge, and Wisdom (DIKW) hierarchy and changing variables.

This structure and other similar ones have been generalized in the literature as the DIKW model (Data, Information, Knowledge, and Wisdom) obeying each of the hierarchical stages of the structure, as presented below in Figure 8.

According to work presented in [50] and in the study presented later [51], the following definitions are essential to understand the hierarchy:

- Data: refers to elemental symbols representing properties of objects, events, activities, or transactions. They are the product of observation or measurement. However, they have no usability or meaning.
- Information: refers to the functional nature of the data. Information is a transformation of the data in an understandable and meaningful format to meet a purpose. It generally answers questions like what, who, and when. Information systems generate, store, retrieve, and process data. Usually, to convert data into information, the processes of classification, rearranging/sorting, aggregating, performing calculations, and selection are required. The authors in [52] noted the importance of the context and the purpose of the information.
- Knowledge: refers to know-how. It is the step that makes it possible to transform information into instructions. Although there is no consensus on its meaning, several authors affirm that knowledge supports decision-making at a primary level [52]. Decision-making requires a combination of common sense and semantic aspects related to interpretation.
- Intelligence and Wisdom: Intelligence is the ability to increase efficiency. Wisdom is the ability to increase effectiveness. The first term is related to growth (of an organization or business), which does not necessarily require added value. Instead, wisdom implies development, which does require added value [50]. The term wisdom involves “human judgment about important, difficult, and uncertain questions associated with the meaning and conduct of life [53].” Some authors relate wisdom to the ability to apply concepts from one domain to new situations or problems and make more in-depth decisions [54].

In addition to the stages of the DIKW hierarchy, the author in [51] mentioned a series of transversal variables that change according to hierarchical stages, as presented in Figure 8. The graph suggests that one step up in the hierarchy requires more human skills to transform the information and give it value (knowledge and wisdom). On the contrary, one step down shows a need for computational helping (information and data).

**Figure 9:**
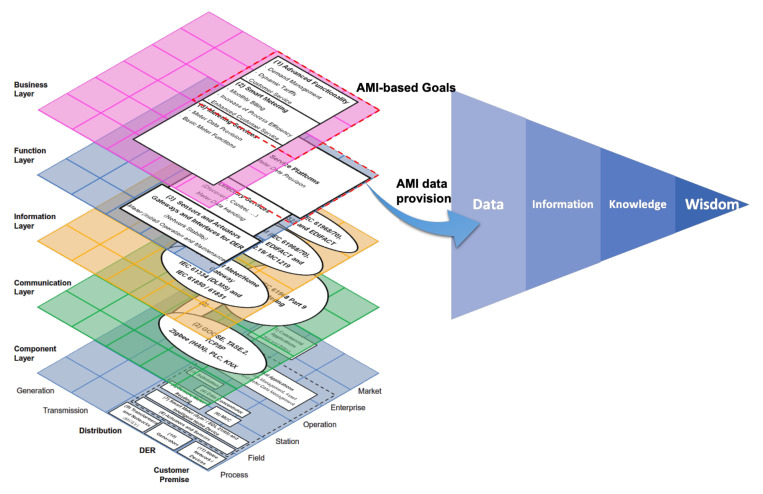
Smart Grid Architecture Model as a data provider for the DIKW hierarchy.

Thus far, we have described two primary components of the framework presented in this paper: AMI framed in the Smart Grid through SGAM and DIKW (Data, Information, Knowledge, and Wisdom) as the evolutionary hierarchy of data. We depict these two components and their relationship below in Figure 9.

Figure 9 presents, on the one hand, the SGAM **architecture** and AMI components in Smart Grids. On the other hand, the DIKW hierarchy sets the goal of transforming data into wisdom. The next step is to establish a **method** that allows this transformation process, which requires consideration of the nature of AMI data, such as the volume, velocity, and variety. These characteristics are the principal components (also known as 3V) of Big Data [55]. Volume involves a growing number of smart meters. Velocity refers to data generation at shot time intervals. Variety refers to the different platforms where data may come from, depending on the application: smart meters and external sources, such as weather databases or Geographic Information Systems (GIS).

As we stated before, according to the application and the nature of the data sources, we can talk about “Big” Data or just “Data”. However, Data Analytics processes, in global terms, involve a general methodology that can be applied. The need to use Big Data techniques (due to the AMI data nature) and Data Analytics (due to the data transformation needs) is evident. To meet these needs, the National Institute of Standards and Technology (NIST) proposed a reference framework for developing Big Data projects [56].

The NIST model always involves a Data Analytics stage. Although the model proposed by NIST was initially for Big Data applications, the following section shows how we can use some components when dealing with Big Data (Big Data Analytics) or just Data (Data Analytics).

#### 2.2.2. Nist Big Data Reference Architecture

**Figure 10:**
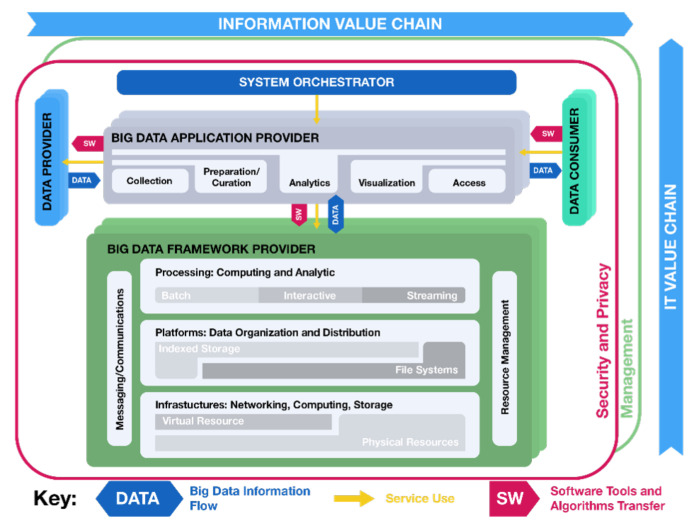
NIST Big Data reference architecture [57].

Reference architectures generally serve as a template for developing solutions in an orderly manner in a specific field and may be used for comparison and alignment purposes. The architecture settled by NIST has been developed by bringing together common elements found in different documented case studies around the world. The reference architecture presented in Figure 10 also includes general considerations on Data Analytics, its implications, and requirements [57].

The reference architecture proposes five primary roles.

- System Orchestrator: defines and integrates the required data application activities into an operational vertical system. It provides the overarching requirements for business ownership, governance, data science, and system architecture.
- Big Data Framework Provider: establishes a computing framework to execute specific transformation applications while protecting the privacy and integrity of data. Resources or services used by the Data Application Provider: Infrastructure framework (networking, computing, storage, environmental), Data platform (physical storage, file systems, logical storage), and Processing (software support for applications). This stage is the most sensitive to the nature of the data to be processed. A Big Data Analytics application may require a more sophisticated computing framework than a Data Analytics application.
- Data Provider: introduces new data (generally, raw data) or information sources into the Big Data system, either online or offline. It is also responsible for data persistence (hosting), data scrubbing (remove PII–personally identifiable information), metadata (for history and re-purposing), policy for others’ access to data, and query without transferring (sometimes).
- Data Consumer: includes end-users or other systems that use the results of the Big Data Application Provider: search and retrieve, download, analyze locally, and reporting and visualization.
- Data Application Provider: executes a life cycle to meet security and privacy requirements. It also develops System Orchestrator-defined requirements, mechanisms to capture data, preparation, analytics (discovery for finding value in big volume datasets), visualization (exploratory, explicatory, or explanatory), and access to the results of the data system. Again, it is essential to note that this life cycle is applicable, in general terms, to both Big Data Analytic and Data Analytics applications.
  Big Data Analysis differs from Data Analysis when it includes the volume, velocity, and variety characteristics of the data under process. Here, we refer to it when we take the AMI data from the function layer (data from smart meters) in the SGAM and the application requirements. In this life cycle, the information is collected, prepared, analyzed, visualized, and accessed.

In addition, the NIST reference model mentions five general stages of this life cycle for the Application Provider:

- Collection: This stage is responsible for connecting to the Data Provider and extracting the data. Such data may be available from various sources. We can refer to this stage as the “extraction” portion of the ETL (Extraction, Transformation, and Load) cycle [58].
- Preparation: At this stage, we carry out the necessary tasks to make the data usable and ready to be analyzed. It includes tasks, such as data validation, cleaning, outlier removal, and standardization. It corresponds to the "transformation" portion of the ETL cycle.
- Analytics: This stage is where we implement all the techniques and algorithms necessary to meet the analysis goal specified by the application. It includes different algorithms and statistical or machine learning approaches. This stage is as complex as defined in the analysis requirement.
- Visualization: In this stage, we prepare the elements resulting from the analytics’ stage and present them to the Data Consumer. Visualization can consist of simple reports or even interactive applications for the end-user.
- Access: This stage is closely related to the visualization stage. It is responsible for giving the required access to the correct user. It can be web services based on access roles or any approach that allows each user, from their role in the application (e.g., manager, operator, or supervisor), to access the results they require.

These five stages generally describe the transformation of data into knowledge that end-users can use. However, other authors have proposed an extended cycle [59] that seeks to “organize the activities and tasks involved with acquiring, processing, analyzing, and re-purposing data.”

**Figure 11:**
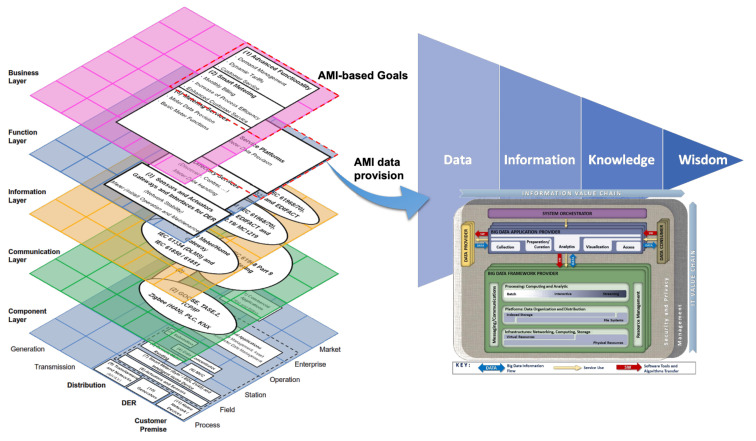
SGAM as data provider architecture for DIKW hierarchy, and NIST model as data transformation methodology.

In any case, whether with the five steps proposed by NIST or the nine of the extended cycle presented in [59], the goal of this stage is to transform the data into usable results through a defined purpose and the use of data analytic techniques and algorithms. Next, Figure 11 adds the NIST reference model as a new element of the framework proposed in this paper.

The framework proposed in Figure 11 presents a relationship between the **architecture** business/function layers of the AMI deployment over SGAM. In turn, the reference framework proposed by NIST acts as a **method** that allows the transformation of AMI raw data into knowledge to advance the hierarchy proposed in the DIKW approach.

### 2.3. The Binding Element: Human Expertise and Skills

In addition to the elements presented in Figure 11, the framework requires a component capable of transforming the knowledge generated from AMI data into a much complex, valuable category in the DIKW hierarchy: wisdom. This last transformation step requires, as mentioned above, human expertise and skills for decision making and judgment about essential decisions and actions [53]. This expertise allows the adaptation of concepts from one domain to new situations [54].

**Figure 12:**
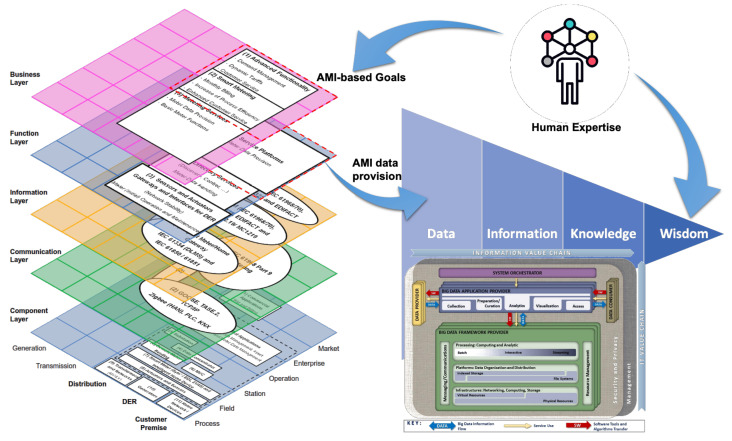
Components and relationships of the proposed framework: SGAM (data provision architecture), DIKW hierarchy (data evolution goal), NIST (methodology to transform data into knowledge), and Human expertise (binding element to transform knowledge into wisdom).

In this way, human expertise plays a crucial role in the business layer of AMI over SGAM by defining goals and objectives and transforming knowledge into wisdom from the DIKW vision. This human expertise, as a new component completing the proposed framework, is shown in Figure 12.

**Figure 13:**
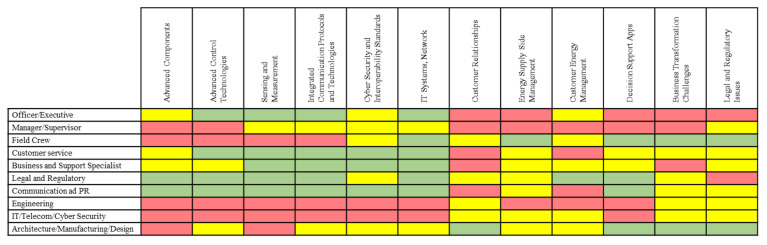
Smart Grid Jobs and Skills Matrix.

West Monroe and the Illinois Institute of Technology conducted a study focused on addressing the US national workforce challenge representing the evolution of power grids into Smart Grids. The study focused on identifying the jobs impacted by the Smart Grid, capturing the level of Smart Grid impact on these jobs. The study defined critical Smart Grid skills requirements and evaluated the current training opportunities to address Smart Grid workforce skill requirements. One of the most relevant results of the report is the Smart Grid Jobs and Skills Matrix specification, as presented in Figure 13 [60].

Figure 13 presents the level of competence required for each job (left) and each Smart Grid skill area (top). The green level involves awareness and understanding of the relevance of the job in the industry. The yellow zone demands knowledge of topics and solutions. At this level, competency of concepts is related to job responsibilities at an intermediate level. The red level requires expertise and mastery (wisdom) of topics and solutions. This expertise is wholly related to the established responsibilities. Therefore, engineering and IT roles are the most related to skills at a level of expertise. Such an idea suggests that interdisciplinary training is an evident need within the evolution of electrical networks context, including AMI.

The Washington State University extension program prepared a report for the Pacific Northwest Center of Excellence for Clean Energy titled “Smart Grid Skills for the Energy Workforce.” This report condensed the opinions of several people involved in smart grid upgrade projects. The objective was to describe the impact of smart grid technology implementation on energy employees’ knowledge, skills, and ability requirements. The study also sought to determine what are some of the significant implications for the education and training of current employees and new hires. According to the reported results, “there was a heightened need for employees who can envision how their work affects—and is affected by—the larger system within which they must operate [61]”.

In general, the report indicated that the new generation of electrical networks requires personnel with system thinking capabilities. This term refers to the vision of activities from two perspectives: operating perspective, related to technical activities, and philosophical perspective, associated with considering the impact of activities on other activities.

The study also indicates that network modernization requires employees with interdisciplinary training and skills to perform in different situations, synthesize information, and have perspectives from different fields. Such a set of skills is called functional knowledge. Among the interdisciplinary areas with the highest value were identified: knowledge of information technology, communications, computer programming, finance, business management, and consumer behavior.

One of the most critical skills highlighted by the study is programming skills and the need for Big Data Analysis and Management training. The results emphasize both the knowledge of how this transmission system works (substations, meters, and general operation) and the programming structural mind-frame required to relate the operation to information systems, communication, data life cycle, among others. The non-technical skills reported in the study grouped project management, interdisciplinary exposure, and understanding customer behavior stand out [61].

## 3. Framework Validation Results: A Case Study

We performed a previous validation of this framework with an exploratory case focused on Big Data and Data Analytics. For that study case, we focused on the electricity consumption data analysis from smart meters in London from 2011 to 2014. We obtained more than 670 million data warehouse records to be processed and analyzed by consolidating different data sources. The Big Data Analytics developments implemented in that previous study case are load forecasting, customer clustering, and modeling and identifying atypical consumption. We already detailed the implementation and results of the case study in a previous number of this Sensors Journal [41].

The case study described in this article was not so focused on Big Data as the previous one but more on Data Analytics due to the volume of records available. For this case study, we implemented each component of the proposed framework for a load forecasting application in a Colombian Retail Electricity Provider (REP). The REP participates in 30 markets associated with two companies belonging to the same REP. Each market corresponds to a city where one of the companies of the REP operates. In this case study, we assigned the names for the companies as Company 1 and Company 5.

Currently, the statistical department of the REP forecasts the energy consumption of its clients as a whole. However, this department found that when they disaggregate the forecasting by each market (city), the Mean Absolute Percentage Error (MAPE) is 38%. The objective of the case study is to implement a pilot project to improve the demand forecasting of the REP’s customers, grouped by market, i.e., by cities in 12-h time intervals (a.m. and p.m. intervals). As it is a preliminary pilot model, the implementation time for this case study was three weeks.

### 3.1. SGAM Business and Function Layers

As in the SGAM presented in Figure 1, the problem described previously defines the goal of the study case, which is the role of the business layer: an application to improve the load forecasting of the company disaggregated by market or city. After the goal definition of the case study, the function layer must guarantee the functionality of the platforms so we can meet the goals defined in the business layer.

According to [43], this layer, for the AMI case, includes service platforms referring to the preparation of the meter data for different roles like distribution network operator, DER operator, or customer. In this sense, the REP gave us access to the data provided by two of its service platforms. The first platform (smart meter data platform) delivers data related to the metering infrastructure system, i.e., data from smart meters installed at each customer premise.

The second platform (customer data platform) displays client-related information. Thus, the goal defined in the case study corresponds to the SGAM business layer. Correspondingly, the data provider platforms to the SGAM function layer. With the components of the business and function layers identified, it is possible to go to the data transformation stage, as indicated on the right side of Figure 12 and depicted in Figure 10.

### 3.2. Transformation of Data into Information and Knowledge

According to the DIKW model [51], we start from raw data (obtained from data platforms in the SGAM function layer) to transform it into knowledge using the reference framework proposed by NIST [62].

- System Orchestrator. The authors and the REP development team fulfilled this role.
- Data Framework Provider. Universidad del Valle provided data storage and processing infrastructure with a cluster with the following capabilities: 11 TB HDD, 138 CPU cores, 384 GB RAM, 23,808 CUDA Cores/176 GB GPU.
- Data Provider. The service platforms mentioned in the SGAM function layer fulfill the role of the data provider. This role links the architectural (SGAM) and the methodological component (DIKW + NIST model) of the framework proposed in this work.
- Data Consumer. The development department and the CEO of the REP assumed this role. They are the end-users of the results of the application.
- Data Application Provider. We developed all processes related to the Data Analytic life-cycle, involving steps like data collection, preparation, curation, analytic, visualization, and access. This role is the one that ensures transformation from raw data to knowledge and covers the data life cycle presented before.

#### 3.2.1. Data Collection and Preparation

The first transformation of data is its conversion into information [51]. To do this, we accomplished the first two tasks as Data Application Provider: data collection and data curation/preparation, as presented in Figure 10. We completed these tasks by building an Extraction-Transformation-Load (ETL) stage [63], creating some functions, and using others from the Pandas Python library [64].

*Extraction.* Using the functions from the Pandas library, we had access to the two data platforms mentioned above, which provided us with historical energy consumption data from 1 January 2018 to 27 November 2019, and customer data. The smart meter data platform delivered one file for each month of historical data. We had access to a total of 1,949,337 records with active and reactive energy measurements hour by hour. For the case study, we only considered active energy consumption. Fifty-six fields composed the data, including customer ID, date, market (city), REP Company, and active and reactive energy measurements for each hour. The customer data platform provided data from 3534 customers and their corresponding characteristics. Eleven fields composed the data, including customer ID, economic activity, contract-related information, electrical installation information, and market (city) for each customer.

*Transformation.* We developed transformation functions using the Pandas library to build a data warehouse, filtering corrupt/incomplete records for both customer and consumption records. We only considered border (total) measurements and grouped consumptions by markets, not by customers, as requested in the REP goal. The smart meter data platform delivered raw data in a matrix-like dataframe with the 56 fields mentioned above. However, once we grouped the data by market, we made 30 time-series dataframes with only five fields: Company ID of the REP, market (City), timestamp (one-hour intervals), type of day: business day, weekend, or holiday, and active energy consumed at each time interval.

*Load.* At this stage, we stored the dataframes in a warehouse with only the necessary information to continue with the required analysis. Table 1 presents the number of records of each dataframe corresponding to the time-series consumption of each market between 1 January 2018, to 27 November 2019 at one-hour intervals.

In this way, we transformed almost 2 million records of raw data into information represented by only 404,401 records grouped into 30 dataframes necessary to run the load forecasting algorithms required for the case study. The next level of DIKW evolution is the transformation of information into knowledge. This transformation corresponds to the tasks of data analytics, visualization, and access presented in the NIST Big Data framework as part of the role of Data Application Provider.

#### 3.2.2. Data Analytics

For this task, we chose a machine learning regression library called XGBoost, available for Python [65]. The name XGBoost stands for “Extreme Gradient Boosting,” a supervised learning algorithm based on gradient boosted trees [66]. The advantage of these regressors is the low computational cost required in assembling several weak learning entities to form a strong entity capable of learning and performing regressions or classifications [67].

We can describe a target $y_{i}$ to be a function of $x_{i}$ inputs as:

(1) $${\hat{y}}_{i}=\sum_{j}\theta_{j}x_{ij}$$

where ${\hat{y}}_{i}$ is the prediction from the input $x_{ij}$, and $\theta_{j}$ is the set of parameters or the undetermined part of the model that needs to be learned by training. The task of training the model is to find the set of parameters $\theta_{j}$ that best fit the training data $x_{ij}$ so ${\hat{y}}_{i}$ can match the target $y_{i}$.The objective function consists of two parts: training loss and regularization term:

(2) $$Fit\left(\theta \right)=L\left(\theta \right)+\Omega \left(\theta \right)$$

where *L* is the training loss function, and Ω is the regularization term. The regularization term controls the complexity of the model, which helps to avoid overfitting. In XGBoost, the classifier and a special assembly of several decision trees define the objective function. The tree ensemble model consists of a set of Classification and Regression Trees (CART). A CART is different from decision trees, in which the leaf only contains decision values. Each leaf has a score in CART, which provides more in-depth information beyond a simple classification [68]. Usually, a single CART is not strong enough. Instead, an ensemble model is better, which sums the prediction of multiple trees together. We can describe the prediction model using CART ensembles as:

(3) $${\hat{y}}_{i}=\sum_{k=1}^{K}f_{k\left(x_{i}\right)},\;\;\;\;f_{k}\in F$$

where *K* is the number of trees, $f_{k}$ is a function in the functional space F, and F is the set of all possible CARTs. The objective function to be optimized is given by

(4) $$Fit\left(\theta \right)=\sum_{i}^{n}L\left(y_{i},{\hat{y}}_{i}\right)+\sum_{k=1}^{K}\Omega \left(f_{k}\right).$$

XGBoost defines the regularization term as:

(5) $$\Omega \left(f\right)=\gamma T+\frac{1}{2}\gamma \sum_{j=1}^{T}w_{j}^{2}$$

where *w* is the vector of scores on the leaves, *T* is the number of leaves, and γ specifies the minimum loss reduction required to do a split. A leaf only splits when the resulting split gives a positive reduction in the loss function. λ is the regularization parameter or penalty term, which determines how much to penalize weights or scores. The logic described by the previous equation was compiled in [65] in the XGBoost algorithm. The implementation principle is learning a behavior (target) from specific characteristics or features (inputs).

For this case study, the target is electricity consumption, and the features are all characteristics that might lead to such consumption. We proposed the features that supported the model as of two types: instant and historical. On the one hand, instant features are those associated with the timestamp of each record as the hour of the day, day of the week, whether it is weekend or not, whether it is a holiday or not, quarter, month, week, and day of the year.

Instant features also can be related to energy consumption, as the average weekday consumption and the hour average consumption according to the timestamp of each record. On the other hand, the historical characteristics refer to energy consumption in previous times, if they influence current consumption behaviors. For the case study, we included historical consumption up to one week before the time stamp indicated in each record. We proposed two configurations for XGBoost regressors. The first one considers only instant features.

The consumption habit may be influenced only by this type of feature. For example, the case of a factory that only operates and produces merchandise on specific days of the week and only at certain hours of the day. These instantaneous characteristics (day and time) directly influence the factory’s energy consumption. The second configuration includes, in addition to the instant features, the historical features. Depending on the activity of a customer, their immediately previous consumptions can influence later behavior. For example, consider the case of a user who wants to keep his consumption within a specific range; if that customer had a high energy consumption during the first days of the month, he might want to reduce it in the subsequent days so as not to exceed any consumption limit.

As each market needed its model, we trained two regressors for each market (one for each available configuration). We used 80% of the available information to train them and evaluate their performance. Although the initial application requirement was prediction at 12-h intervals, given the granularity of the data, regressors were trained to make hourly predictions, that is, with greater detail. We selected the MAPE between the actual consumption and the predicted energy consumption for each hour of the day as performance measurement.

For each market, the algorithm chose the regressor with the configuration that presented the lowest MAPE performance in the training stage. Table 2 presents the MAPE measured every hour for each market and company in the third column. As observed in Table 2, the markets Putumayo, Tolima, and Tuluá from Company 1 and Costa Caribe, and Tolima from Company 5 have a higher average percentage error. By looking at the number of records available according to Table 1, we found that due to the low data availability, the training and learning process of the regressors did not perform well. Therefore, the prediction results had a higher error rate.

#### 3.2.3. Data Visualization and Access

**Figure 14:**
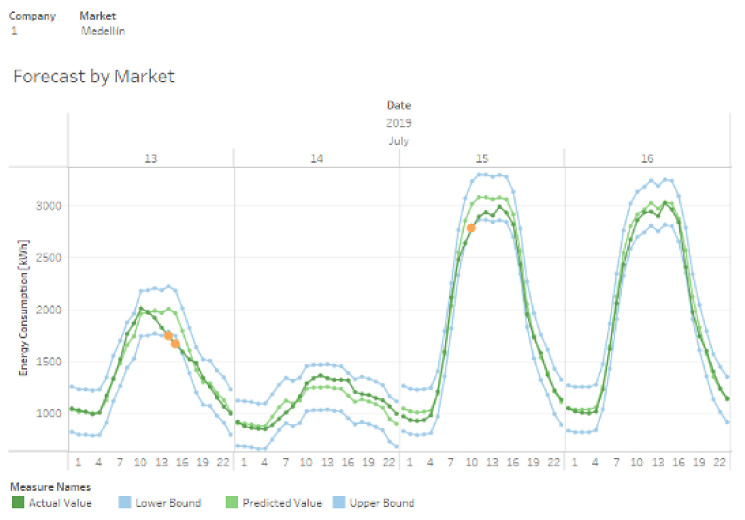
Snapshot of the forecasting dashboard for the Medellín market, Company 1: real vs. predicted values and upper/lower confidence intervals.

We used Tableau dashboards for the result visualization. Figure 14 presents a graph with the forecasting results for the Medellín market on 13–16 July 2019. The blue lines represent the upper and lower confidence intervals. The dark green line represents the actual energy consumption for each market, while the light green line represents the forecasted value. The orange dots on some data represent points where the consumption was outside the expected range. These points probably represent an anomaly consumption. The goal of the case study was to improve consumption forecasting at 12-h intervals, considering that REP has a MAPE of 38% when dis-aggregated by each market.

To establish the same comparison scenario, we grouped the hourly consumption predictions into 12-h intervals (a.m. and p.m), as shown in the last column of Table 2. As expected, grouping the measurements and predictions into longer time intervals decreased the average percentage error since the forecast error for some hours of the day may be compensated by other points in the same time interval (a-m. or p.m.). The transformation of data into knowledge using data analytic techniques improved the MAPE from 38% to 8.90% for energy consumption forecasting in 12-h intervals dis-aggregated by company and market.

We also designed some dashboards with descriptive analysis. They do not imply the implementation of any data analytic or machine learning technique. However, this additional visualization aimed to facilitate data consumer access to the information used in the case study from the data provider platform. Once we transform the information into knowledge, this knowledge is now usable and available for the data consumer. In this case study, data consumers are the REP’s development department and the CEO.

### 3.3. Transformation of Knowledge into Wisdom

The stages of the framework implemented up to this point would allow the primary goal initially established in the case study to be met: improving demand forecasting at 12-h intervals for a REP, disaggregated by market. However, the capacities and skills of the REP development team and our research group allowed us to go a step further. The training of professionals from both the REP and our research group was interdisciplinary. Thus, with programming and algorithm implementation capabilities (DIKW hierarchy + NIST framework), we also knew the global context of the operation of a REP within the electricity grid (SGAM).

Beyond the operation, REP’s supervisors also knew about the most recent regulations in the country’s electricity sector. With the ability to understand the implications and results of the implemented framework with a deeper vision, the team experts identified a further need with a more significant impact on the REP related to a new regulation soon to come into force: CREG 100/2019.

According to this regulation, each REP must report to XM their demand forecasts for each market where they operate instead of doing it as a whole. XM is the national entity in charge of the operation of the Colombian electrical system (https://www.xm.com.co (accessed on 7 August 2021)). Each REP should present their predictions daily for each 12-h interval of the day. They may have a maximum prediction error per interval of 4%. Otherwise, XM could penalize the REP according to the error measure in the demand forecasting of each market [69].

At this point, the case study had expert knowledge from:

- Our research team, who in addition to IT and data analytics skills, had a broad context of the operation of AMI systems.
- Operation and supervision team of the REP, who knew in detail the implication of the new regulation and the benefit that our development could represent. They were also in charge of supplying the necessary data to develop the case study and guarantee the security and privacy policies of the REP’s information.
- The REP’s CEO, who knew the economic impact for the company, once the new regulation came into effect. The CREG 100/2019 regulation implies potential financial sanctions in case of exceeding the margin of forecasting error established in the regulation.

The multidisciplinary knowledge of the team and the deeper understanding of the results were evident in the implementation of this pilot. Furthermore, the team provided the CEO with the necessary elements to make a data-driven decision: to make a more significant investment in the implementation of a new business platform. This new business platform would aim to implement the second phase of this pilot to get a more robust forecasting application aligned with the CREG 100/2019 requirements that will come into effect soon. The proposed investment for this platform will generate benefits for both the company and its customers. At the same time, it will promote cooperation between research institutions and private energy companies.

We see how the elements present in the team match the roles that require deeper knowledge to give decision support according to Figure 13: executives, supervisors, engineering and IT teams. This accounts for the importance of the levels of competence/expertise of critical roles in certain specific areas of a Smart Grid, as in this case, informed decision-making. Recalling Figure 8, we can also note how, at these higher levels, human judgment derived from experience and a broad context (both technological and business) has greater value, as opposed to the initial transformations of raw data, where the greatest workload falls. clearly on the computing infrastructure.

**Figure 15:**
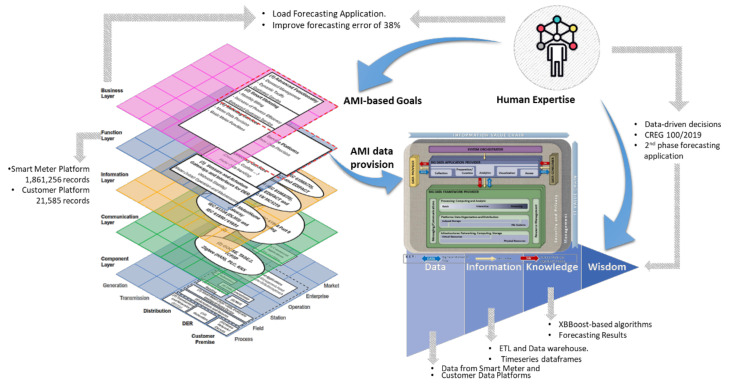
The stages of the case study mapped on the proposed Big Data/Data Analytics framework.

Figure 15 presents each element of the framework depicted in Figure 12 with the elements from the case study presented in this paper. Gray arrows indicate the correspondence of each stage on the proposed framework. The REP team initially defined the goal as a pilot project for load forecasting based on measurements from smart meters. This goal corresponds to a goal in the SGAM business layer.

The expertise and skills of the human team provided enough supplies for these corporate-level decisions. As we presented in previous sections, the ability to apply concepts from one domain to new situations or problems allowed the transformation of knowledge into wisdom and informed decisions that benefit the REP’s work in a broader context. The smart meter and customer data platforms acted as data providers for the SGAM function layer. That same data provision is the initial input of the DIKW hierarchy. Using the NIST framework, data was transformed, first into information and then into knowledge.

Initially, we built a data warehouse implementing an ETL stage to store relevant information for the case study. Later we used XGBoost-based algorithms to implement forecasting models and generate knowledge. We used Tableau dashboards to facilitate the access to results by end-users. Finally, thanks to the human expertise and skills of the team, we reached a greater understanding of the benefits of this application and its possible impact related to new regulations of the electricity sector. This acquired wisdom made it possible to make informed decisions to create new investments to strengthen the REP’s analysis platforms in the second phase of the pilot project.

## 4. Conclusions

The evolution of the electrical grid in Smart Grids opens the way to new infrastructures implementations, including AMI. Its deployment makes available a volume of data that grows as fast as AMI project implementations. This availability of data from Smart Meters (AMI data) requires tools and platforms for its processing, analysis, and use through fields of study, such as Big Data and Data Analytics. In this regard, several authors have presented study approaches and applications. Some applications include AMI data processing tools and their integration with emerging technologies, load profile identification, load forecasting, demand response programs, and loss detection.

The literature shows that several authors have studied Big Data/Data Analytics in AMI and Smart Grids. Some of them proposed different approaches and methods to perform data transformation [7,22,40,70,71,72,73]. However, most of the works only achieved data analysis (methods) with a specific purpose, e.g., load forecasting, loss detection, or load profiling, without a relationship to the global view proposed by SGAM (architecture). This lack of connection implies that, although such works meet an analysis goal from raw data to knowledge, they do not reach wisdom, in the sense that the results are not always applied to new domains or situations to make more in-depth decisions. The most important contribution of this paper is a framework that allows the evolution from raw AMI data to applied wisdom in different areas of a Smart Grid.

This is achieved through a framework that joins the vision of three perspectives: first, an architectural view for the deployment of AMI in the context of Smart Grids; from this architecture, business goals can be defined at the top level, down to the physical components required for AMI operation. The architecture establishes a level where one has access to platforms that act as the source of AMI data. The second perspective involves the transformation of the AMI data. This transformation includes using Big Data/Data Analytics techniques and their life cycle to give value to the data and transform it into knowledge through different available methods. Finally, human expertise and skills appear in the third perspective as binding element of the framework.

This provides a last evolution step from knowledge to wisdom as the ability to include human judgment, reasoning, and higher level of understanding. This superior transformation increases the value of developments related to AMI data. The new generation of electrical networks requires multidisciplinary teams to achieve such a deeper understanding of Smart Grid processes. Likewise, a greater understanding will allow informed decision-making with a global impact that benefits different Smart Grid value chain links.

The implementation of a case study with real data allowed us to validate the framework. The case study shows that all its components play an essential role in achieving results that globally benefit the operation of a company in the electricity sector, in this case, a REP. The smart meter and customer data platforms acted as data providers for the SGAM function layer. That same data provision was also the input of the DIKW hierarchy. We used the NIST method to transform raw data into knowledge: fisrt, we implemented and ETL and a data warehouse; later, we used XGBoost for perform forecasting. We used Tableau dashboards to deliver results to end-users. The pilot project implemented in this case study reduced the forecasting MAPE from 38% to 8.9%, considering the short development time.

Future investments, deployed from the transformation of raw data into wisdom, will further improve the results of this application. Finally, using the human expertise and skills of the team, we reached a greater understanding of the benefits of this application and its possible impact related to upcoming regulations in the electricity sector. This human judgment was the product of the support that our team was able to provide to make an informed decision. This shows that our application case study not only delivered knowledge (as a forecasting model) but that a higher level of concept application was reached with a wider impact for the benefit of the REP.

As future works, we propose the application of this framework in other scenarios of a Smart City, taking advantage of the great availability of data from various platforms: energy efficiency, smart mobility, smart metering (water, gas), and smart billing, among others. This could mean an optimization of resources and a change in the operating dynamics of the entire Smart City scheme. The global objective of a Smart City should be the optimal and efficient operation of a system of systems where the availability of data in real time has become a differentiating factor.

In addition, the inclusion of various artificial intelligence techniques can be considered to broaden the spectrum of data transformation. We achieved this by including other sources of information, such as data from social networks, thus, allowing users of a smart city to be a stronger input in our framework. Some approaches to dealing with this type of data could be Natural Language Processing (NLP) or sentimental analysis. Although the paper leaves open the possibility of including different types of data processing, there is still room for subsequent validations that include distributed processing platforms or case studies more focused on data privacy and security.

## Figures and Tables

**Table 1:** Dataframes and records in the warehouse.

Company ID	Market	Records for Market
1	Antioquia, Bogotá, Boyacá, Caldas, Cali, Cartago, Casanare, Cauca, C. Atlántica, C. Caribe, Medellín, Nariño, Quindío, Valle del Cauca.	16,704
1	Cundinamarca	10,176
1	Putumayo, Tolima	9168
1	Santander	12,481
1	Tuluá	9504
5	Antioquia	12,768
5	Bogotá	12,936
5	Cali	14,616
5	Costa Atlántica	3528
5	Costa Caribe	1968
5	Entrerrios	6552
5	Medellín	13,440
5	Pereira	1272
5	Putumayo	9312
5	Tolima	8112
5	Valle del Cauca	13,272
	**Total Records in Warehouse**	**404,401**

**Table 2:** MAPE by one-hour and 12-h intervals for each company and market.

Company ID	Market	MAPE by hour	MAPE by 12-h
1	Antioquia	5.79%	3.86
1	Bogotá	4.66%	3.91%
1	Boyacá	33.16%	15.16%
1	Caldas	30.30%	13.30%
1	Cali	4.45%	3.57%
1	Cartago	6.24%	3.85%
1	Casanare	0.01%	0.02%
1	Cauca	16.71%	10.61%
1	Costa Atlantica	17.43%	12.33%
1	Costa caribe	7.14%	4.88%
1	Medellín	5.60%	4.20%
1	Nariño	6.24%	4.57%
1	Putumayo	101.01%	41.69%
1	Quindío	12.56%	5.35%
1	Santander	24.10%	14.77%
1	Tolima	74.71%	32.87%
1	Tuluá	54.30%	27.03%
1	Valle del Cauca	6.33%	4.04%
5	Antioquia	9.58%	8.01%
5	Bogotá	7.82%	6.46%
5	Cali	7.69%	6.42%
5	Costa Atlantica	15.38%	10.57%
5	Costa Caribe	140.56%	62.19%
5	Entrerrios	17.21%	13.66%
5	Medellín	9.78%	8.76%
5	Pereira	17.75%	11.97%
5	Putumayo	10.72%	5.54%
5	Tolima	64.61%	7.87%
5	Valle del Cauca	9.33%	6.44%
**MAPE for all forecasts**		17.45%	8.90%

## Data Availability

Not applicable.
